# Diagnosis Based on Population Data versus Personalized Data: The Evolving Paradigm in Laboratory Medicine

**DOI:** 10.3390/diagnostics14192135

**Published:** 2024-09-25

**Authors:** Abdurrahman Coskun

**Affiliations:** Department of Medical Biochemistry, School of Medicine, Acıbadem Mehmet Ali Aydinlar University, 34752 Istanbul, Turkey; coskun2002@gmail.com; Tel.: +90-500-40-69

**Keywords:** decision limits, personalized laboratory medicine, personalized reference interval, reference change value, reference interval

## Abstract

The diagnosis of diseases is a complex process involving the integration of multiple parameters obtained from various sources, including laboratory findings. The interpretation of laboratory data is inherently comparative, necessitating reliable references for accurate assessment. Different types of references, such as reference intervals, decision limits, action limits, and reference change values, are essential tools in the interpretation of laboratory data. Although these references are used to interpret individual laboratory data, they are typically derived from population data, which raises concerns about their reliability and consequently the accuracy of interpretation of individuals’ laboratory data. The accuracy of diagnosis is critical to all subsequent steps in medical practice, making the estimate of reliable references a priority. For more precise interpretation, references should ideally be derived from an individual’s own data rather than from population averages. This manuscript summarizes the current sources of references used in laboratory data interpretation, examines the references themselves, and discusses the transition from population-based laboratory medicine to personalized laboratory medicine.

## 1. Introduction

The diagnosis of diseases is a complex procedure based on multiple parameters obtained from different sources, including patient history, physical examination, laboratory findings, radiological findings, histopathological examinations, and more. Among these parameters, laboratory findings are crucial as they form the basis of clinical decisions [1,2]. The interpretation of laboratory data is a comparative procedure that requires reliable references for accurate interpretation. Different references such as the reference interval (RI) [3,4,5], decision limits (DLs) [6], action limits (ALs), reference change value (RCV) [7], etc., are used for the interpretation of laboratory data depending on the data type, the clinical situation of the patients, and the type of diseases [8]. However, these references are derived from population data but are used to interpret individual laboratory results rather than population data [9]. The difference between the sources of references and the data that they interpret raises questions about the reliability of the references and, consequently, the accuracy of interpreting laboratory data. Since diagnosis is the first step of medical practice, its accuracy is crucial for subsequent steps such as effective treatment, monitoring, and evaluating the prognosis of disease. Therefore, reliable references should be considered a priority in medical practice. Since there are no two identical humans on our planet, if possible, the references used to interpret laboratory data should be obtained from the individual’s own data [10]. However, this is not as easy as obtaining references from the population data due to several limitations, such as the limited number of data points used to estimate personalized references, as well as the timing and collection of sampling [11]. In this manuscript, I summarize (i) the characteristics of laboratory data, (ii) the diagnosis of diseases using multiple tools including laboratory data, (iii) the source of references based on population and individuals’ data, (iv) the references used to interpret laboratory data, and (v) the evolution of population-based laboratory medicine to personalized laboratory medicine.

## 2. Characteristics of Laboratory Data

**Figure 1:**
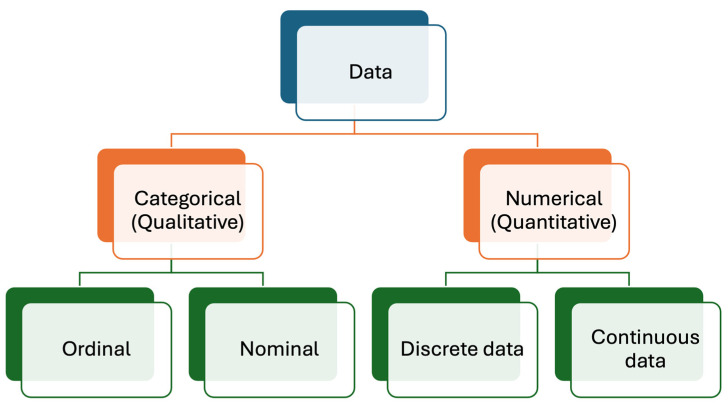
Data types produced in medical laboratories. Numerical (quantitative) data are more common than categorical (qualitative) data.

Medical laboratories produce different types of data, mainly numerical (quantitative) and categorical (qualitative) [12,13], as briefly outlined below (Figure 1).

### 2.1. Numerical Data

This type of data provides information about the quantities of analytes and is one of the most common types of data generated by medical laboratories. This type of data, exemplified by the measurement of analytes like glucose, cholesterol, etc., quantifies results using numerical values. For instance, glucose levels might be reported as 100 mg/dL. In this example, 100 represents numerical data. Numerical data can be categorized as discrete and continuous data, as detailed below.

#### 2.1.1. Discrete Data

Discrete data are the data type of data that used to count and can only take certain values. In laboratory medicine, the number of cells such as leukocytes, erythrocytes, platelets, bacteria, parasites, etc., are expressed as discrete data. Additionally, the number of samples, repeated measurements, patients, and all other countable things are expressed with discrete numbers. Discrete data can take only specific values and are expressed with whole numbers such as 1, 2, 3, etc.

#### 2.1.2. Continuous Data

Continuous data are the primary type used to express measurement results. Mathematically, the primary difference between discrete and continuous data lies in the representation of values. Continuous data can include decimal places, allowing for a more precise expression of measurements. For example, serum potassium levels can be reported as 4.2 mmol/L. The number of decimal places used depends on the significance of the data and the uncertainty of the measurement process.

### 2.2. Categorical Data

They can be classified as nominal and ordinal data. 

#### 2.2.1. Nominal Data

Nominal data are a type of categorical data that are used to label data without any quantitative value, such as female, male, name of people, colors, etc. 

#### 2.2.2. Ordinal Data

Ordinal data are a type of data where the categories have a meaningful order or ranking. Unlike nominal data, ordinal data allow for the comparison of items or ranks, such as first, second, and third, or classifications like none, mild, moderate, and severe.

## 3. Laboratory Data and Statistical Distributions

Laboratory data are not fixed values; they fluctuate due to pre-analytical, analytical, biological, chronobiological, and lifelong variations. Therefore, even for an individual, the measurement results of repeated samples taken at different times are not identical; they exhibit a distribution. The characteristics of these distributions are determined by the factors influencing patients and data variation.

**Figure 2:**
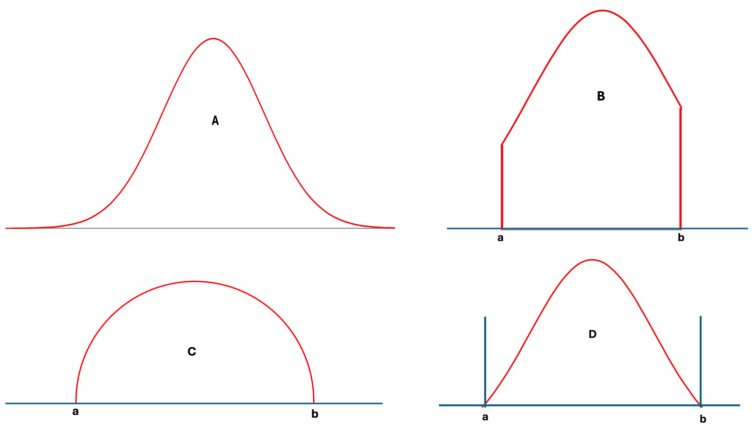
Various distribution types used in statistical analysis: normal distribution (**A**), truncated normal distribution (**B**), semicircular distribution (**C**), and a hypothetical distribution with lower and upper limits (**D**). For laboratory data, (**D**) and its skewed derivatives appear to be more realistic.

In statistics, many different distribution types have been identified and analyzed in detail [14]. However, only a few well-known distributions, such as the normal, log-normal, and chi-square distributions, are frequently used in analyzing laboratory data. These distributions, however, are not always well-suited for laboratory data, particularly from healthy individuals, as such data have lower and upper limits determined by physiological mechanisms. During disease, the control of these physiological mechanisms weakens, and the range of laboratory data widens compared to that of healthy individuals [15]. Nevertheless, these data still have upper and lower limits. In other words, extreme values, which are part of normal or log-normal distributions, etc., are not applicable to individual data, and the measurement results of any measurand cannot be infinite or negative. For example, repeated measurements of blood pH do not contain values like 1.0 or 20, because these levels are not compatible with human metabolism. Therefore, according to a normality test such as those of Kolmogorov–Smirnov or Shapiro–Wilk, if the measurement results of samples obtained from an individual are normally distributed, it is important to acknowledge that in practice, the data themselves will never perfectly align with every data point present in the idealized normal distribution. Ideally, truncated normal, truncated log-normal, truncated t, semicircular, triangular, bimodal, multimodal, or other distributions with defined upper and lower limits—or skewed forms of these distributions—may be better suited to laboratory data than the classical normal, log-normal, or similar distributions [14,16] (Figure 2A–D).

## 4. Diagnosis of Diseases

The diagnosis of diseases is a complex procedure and usually requires evidence obtained from multiple sources, including laboratory data. Obtaining laboratory data is relatively easy and frequently does not require invasive procedures. Therefore, it is often requested by physicians and, after the patient’s history and physical examination, is the first line of data used for diagnosis. Despite these positive aspects of laboratory tests, for most, the diagnostic power of a single laboratory test is unfortunately not strong. Therefore, physicians usually need additional evidence to confirm the diagnosis, particularly if the results of laboratory tests do not align with the patient’s history and clinical findings. The new evidence may include requesting panels of tests or additional laboratory tests related to the possible diagnosis of diseases or different types of evidence such as radiological imaging tests, pathological tests using microscopic examination of biopsies, genetic analysis, etc., depending on the clinical findings of the patients, the probable diagnosis made by physicians, and the results of laboratory tests. It should be noted that although these additional tests increase diagnostic power and make it easier for physicians to make decisions regarding the diagnosis, they are often more invasive, such as taking tissue biopsies, or potentially harmful, such as the radiation used in radiological imaging. They are also much more expensive than most laboratory tests. Consequently, to increase the diagnostic power of laboratory tests, it is essential to measure and interpret the laboratory test results accurately. The interpretation of laboratory data is a comparative procedure, and for accurate interpretation, it requires reliable references for comparison [17]. Reliable references should be obtained from credible sources using correct mathematical approaches, as detailed below.

### 4.1. The Theory of References for Measurands

It is noteworthy that for a healthy individual, the reference value used to compare laboratory data is an interval rather than a single exact value. The reason for this is the physiological fluctuation (variation) in the concentration of biomolecules around a homeostatic set point (HSP) [17,18]. It should be noted that for an analyte, if the variation around the HSP is obtained from an individual’s own data, it is known as within-person biological variation (CV_P_). However, if it is obtained from the data of a group of individuals, it is referred to as within-subject biological variation (CV_I_). The HSP of different subjects are different, and for an analyte, the variation in HSPs among different individuals is known as between-subject biological variation (CV_G_). The concentration of some biomolecules, such as hormones, is regulated, while other molecules are influenced by production, excretion, or degradation. In any case, it can be observed that there is fluctuation around the HSP for all molecules, and that CV_P_, CV_I_, and CV_G_ are analyte-specific. 

For an analyte, two types of reference intervals can be estimated: the ideal one is the individual specific RI, which is known as the personalized reference interval (prRI). The upper and lower limits of the fluctuation around the HSP, i.e., the limits of CV_P_, are referred to as the limits of prRI. The second one is the population-based RI (popRI), which is derived from the data of the population. The popRI can be estimated from the Gaussian combination of both CV_I_ and CV_G_. 

Currently, in routine practice, the popRIs of analytes are estimated using the measurement results of a single sample obtained from each individual in a group. The number of reference individuals should exceed 120. Although this approach has been widely used since the 1960s, concerns have been raised about the reliability of popRIs derived from this method [19]. Recently, we developed models using the biological variation (BV) data of analytes to estimate both prRIs [8,9,10,11,17,20,21,22] and popRIs [23].

**Figure 3:**
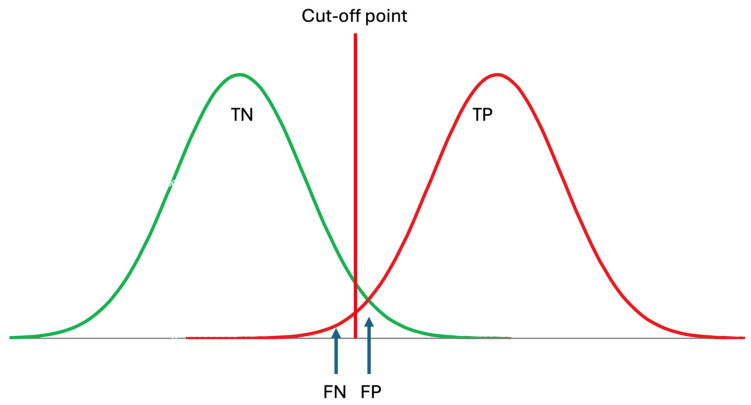
Reference intervals are derived from data obtained from healthy subjects, thereby reflecting the specificity of the measurand, which can be expressed as TN/(TN + FP), where TN represents true negatives and FP represents false positives. TP: true positive, FN: false negative. Although patient data may not be normally distributed, for simplicity, it is assumed to be normally distributed in this figure.

It should be noted that the RI of an analyte is the interval within which the located measurement results are considered indicative of healthy individuals, i.e., those without diseases. Therefore, RIs optimize the specificity of laboratory tests as outlined below [24] (Figure 3).

(1) $$Specificity=\frac{TN}{TN+FP}$$

where *TN* is the true negative and *FP* is the false positive rate predicted by the RI. In the conventional approach, historically, it is accepted that the RI covers 95% of data from healthy individuals, with 2.5% of the healthy population’s data located below the lower limit and 2.5% located above the upper limit. The clinically significant reference limit is the decisive factor in the specificity of the RIs. Therefore, the confidence interval (CI) of the clinically significant reference limit has a significant impact on the reliability of the RIs. It is recommended that the CI of the reference limits should not exceed 0.2 times the range of RI [3].

**Figure 4:**
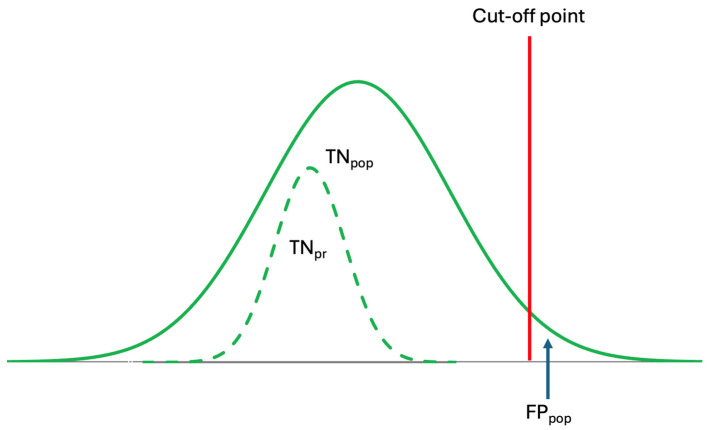
Conventional reference intervals are derived from population data but are used to interpret individual laboratory results. However, because prRIs differ from popRIs, the specificity of popRI is low, leading to misinterpretation. The dashed curve represents the prRI.

In contrast to RI, which represent a range, DL and AL are thresholds estimated from patient data, used for disease diagnosis and medical interventions. Changes in data for healthy individuals occur gradually and are time-dependent, requiring varying durations for diagnosis. The duration needed for changes—from RIs to crossing DLs or ALs—is specific to both the disease and individuals’ health conditions (Figure 4). 

Although population-based reference intervals (popRI) DL, and ALs, are currently being used in clinical practice, in reality, these references are not specific for individuals [10,19]. As shown in Figure 4, the specificity of the popRI for individuals is low and may not be reliable. Therefore, for the accurate interpretation of laboratory data, personalized references should be used, as they are both analyte- and individual-specific.

### 4.2. The Source of References to Interpret Laboratory Test Results

In the real world, the source of scientific knowledge is data; knowledge not grounded in reliable data is blind. Reliable knowledge is built upon reliable data, making it essential to scrutinize both the quality and representativeness of the data for its intended purpose before extracting knowledge from it. Numerous references, such as RIs, DLs, ALs, and RCVs, are used to interpret laboratory test results [8]. These references are chosen depending on the purposes for which the laboratory tests are being used, such as diagnosis, monitoring, and planning of treatment, as well as the types and concentrations/activities of the laboratory data. The sources of all these references are biological samples obtained from the relevant individuals, as outlined below.

#### 4.2.1. Population-Based Big Data

**Figure 5:**
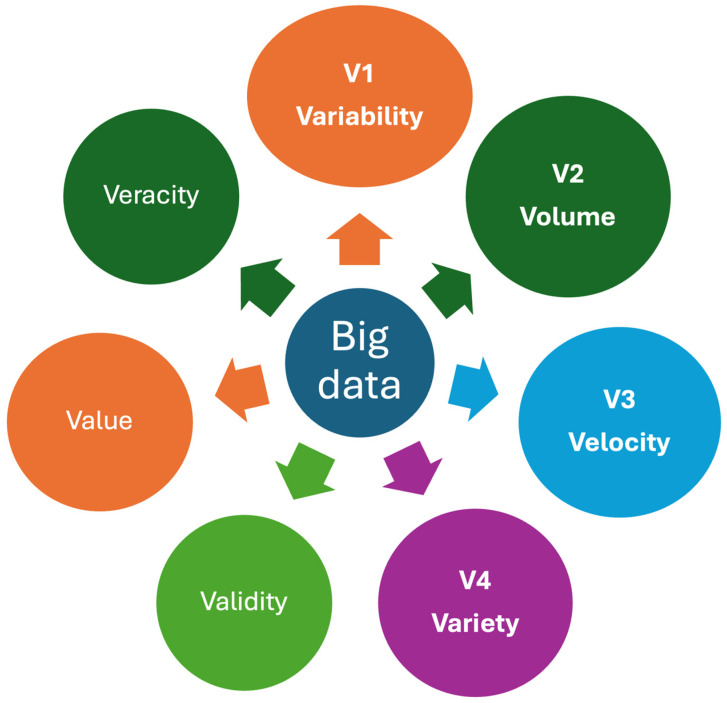
Some important Vs for healthcare big data. Big data is characterized by multiple Vs, which refer to the attributes of complex and large datasets that exceed the capabilities of traditional data processing tools to analyze, interpret, and manage. Variability, a property unique to laboratory data, is included as a new “V” alongside the existing “V”s that represent the characteristics of laboratory big data. Veracity represents the quality, accuracy, and integrity of big data.

Big data refers to complex and large datasets that are beyond the capacity of traditional data processing tools to analyze, interpret, and manage. They are characterized with “V”s, and multiple “V”s have been attributed to big data [25]. Despite multiple Vs, three main Vs, namely volume, velocity, and variety, have been used widely for big data [26,27]. However, it should be noted that the characteristics of healthcare data, particularly laboratory data, differ from industrial or other datasets. Since laboratory data often include repeated measurements from individuals, a new “V” representing “variability”, known as the CV_P_ of the analyte for the individual, can be added to the existing “V”s as a new component (Figure 5). 

Population-based data have both significant advantages and disadvantages for laboratory medicine. The primary advantage is that the volume of data can be substantial. Increasing the volume of data decreases the uncertainty of the precision of statistical models. For example, the mean of a population can be estimated using the mean of a small dataset randomly selected from the population. However, in such cases, the representativeness of this mean for the entire population may be questionable because the uncertainty of the sample mean is inversely proportional to the square root of the number of data points in the sample selected from the population, as formulated below:

(2) $$CI=k\times \frac{SD}{\sqrt{n}}$$

where *CI* is the confidence interval of the mean, *SD* is the standard deviation, *n* is the number of data points in the sample selected from the population, and *k* is the coverage factor depending on the statistical distribution of the dataset. It should be noted that the main purpose of using big data is not only to reduce the uncertainty of the precision of statistical models but also to ensure that the selected sample represents the population. Increasing the number of data points enhances the representativeness of the sample for the entire population. This is important particularly in clinical studies. 

Because of biological diversity, attaining a completely homogeneous population is not achievable. Therefore, despite statistical homogeneity, each population should be considered heterogenous. The degree of heterogeneity can vary depending on the composition of the population, but typically, it is higher than zero. Thus, increasing the number of data points in the sample can be helpful in detecting minor differences or rarely observed serious clinical situations within the population.

Since references are used to categorize individuals based on their health status, data from both healthy and diseased subjects are necessary to derive reliable references.

##### Population-Based Big Data of Healthy Subjects

Obtaining big data from healthy subjects in the real world is not as easy as expected. Barriers such as ethical, technical, and economic issues limit the collection of big data from healthy populations. Therefore, this type of data is usually obtained from hospitals using some statistical extraction method to exclude the data of non-healthy subjects. Hospital data include both healthy subjects and patient data, and although some statistical methods have been developed to extract data from healthy subjects, it remains challenging to do so. As a result, the quality of data from healthy subjects obtained from hospitals or medical laboratories may not always meet the desired standards.

While popRI can be estimated from the data of at least 120 healthy individuals using the direct method, population-based big data of healthy subjects obtained from hospitals or medical laboratories are commonly used to estimate RIs using the indirect method. Moreover, to derive reliable references, data from only healthy subjects are not adequate because some references, such as DL and AL, which are crucial for clinical decisions, are derived from patient data rather than from the data of healthy individuals as previously mentioned.

##### Population-Based Big Data of Diseased Subjects

References based on data from healthy subjects have limitations when used to diagnose certain diseases or evaluate their severity. For example, serum glucose levels are used for diagnosing diabetes mellitus (DM). For healthy adult subjects, the upper limit of the RI for glucose is not the cutoff for diagnosing DM. According to American Diabetes Association (ADA) guideline, the cutoff value for diagnosing DM with fasting plasma glucose is 126 mg/dL [28]. In clinical practice, there is often a gap between the limits of the RI and the DL for diseases. For example, the UL of the RI for an analyte typically represents the 97.5th percentile of healthy subjects, meaning that only 2.5% of healthy individuals have values above this threshold. Consequently, the UL of the RI can be considered the boundary for measurement results from healthy subjects. Therefore, for accurate disease diagnosis, data beyond the RIs are needed to estimate the DLs, and such data can only be obtained from diseased subjects. 

For the diagnosis, monitoring, or medical intervention of diseases, clinical outcome data are valuable, but they may vary depending on the clinical trial and the methods used to obtain them. The first The European Federation of Clinical Chemistry and Laboratory Medicine (EFLM) Strategic Conference updated the hierarchy of models, previously known as the Stockholm Consensus, now referred to as the Milan Criteria. These criteria are used to establish analytical performance specifications [18]. In the Milan Criteria, the impact of analytical variation on clinical outcomes is recognized as the primary criterium for establishing the analytical performance specifications for analytes. Therefore, patient data used to estimate reference values, particularly for DLs, should be derived from accurate sources and methods.

It should be noted that RIs evaluate the specificity of laboratory tests, i.e., the ability to confirm the absence of a disease, but not their sensitivity, i.e., the ability to detect the presence of a disease. Although plenty of data from diseased subjects are available in medical laboratory information systems (LIS), these data belong to patients with various clinical situations and do not have the same quality. Therefore, these data should not be used in estimating DL, AL, and other cutoff points without proper evaluation and filtration. 

For an analyte, at least theoretically, patients’ data are more heterogeneous than the data of healthy subjects. In healthy subjects, in addition to CV_I_, CV_G_ is the main variation. However, for patients’ data, there are additional factors that increase the variation between individuals, such as the severity of diseases, treatment protocols, host response, etc. Since the concentration of biomolecules is under physiological control, pathological situations adversely affect this control. In such cases, due to weak physiological control, fluctuations in biomolecules increase, and the HSPs shift from their normal levels. The control of these fluctuations and the HSPs weakens depending on the severity of the disease and the effectiveness of the treatment. Increased or decreased concentrations of biomolecules are used for the diagnosis of diseases, such as DL. However, fluctuations in biomolecule concentrations have not yet been utilized for the diagnosis or monitoring of diseases.

DLs should be derived from less variable data, and it should be noted that increasing variability decreases the reliability of DLs. To reduce these variabilities, the DLs of analytes should ideally be estimated based on individuals’ own data. However, this is not easy in practice, and there are several barriers to using individuals’ own data to estimate the DLs of the analytes, which is essential for the accurate diagnosis of diseases. Using individuals’ own data is not the solution to all problems related to reference values because, in the real world, it is not possible to collect hundreds of data points from each individual. Therefore, statistical algorithms based on small datasets should be used to estimate references for individuals.

#### 4.2.2. Individuals’ Small Datasets

Conventional statistics is shaped based on the population–sample paradigm [9]. Populations are defined by large datasets, while samples consist of a smaller, randomly selected subset of data points from the population, which allows for inferences to be made about the broader population. The representative capacity of the sample for the population is the critical point, and some statistical parameters, such as confidence interval and uncertainty, can be used to express this representativeness. To increase the representativeness of samples for the population, the number of data points in the samples is increased. 

In contrast to the population–sample paradigm, the characteristics of a small dataset obtained from individuals differ, as outlined below.

Although, theoretically, the number of data points for a healthy individual can be as high as a population dataset, in practice, this is not possible. In reality, the number of data points for an analyte for a healthy individual is limited, typically around 10 or even less, and rarely higher than 30. This is because for each data point, the individual must go to a hospital, the samples must be obtained from the individual, and the analyte must be measured, and this is not realistic in the real world to increase the number of measurement results to a high level. In other words, the classical dual population–sample paradigm is not applicable to small-sample-size groups, and we need a small data paradigm particularly for personalized medicine [29]. However, despite the small sample size, valuable information can be extracted from a small dataset using appropriate tools [29,30,31,32]. For individuals, references based on their own data derived from a limited number of data points are often more reliable than population-based references estimated from larger datasets [22] (Figure 4). Because individual data are more homogeneous and specific to each person, references derived from population data may not represent all individuals equally. Therefore, population data may not serve as an ideal reference for the individual. 

### 4.3. Population-Based Big Data and Personalized Laboratory Medicine

Big data can be effectively utilized to extract valuable insights for populations. However, it is not always a reliable source for individuals. Data not obtained specifically from an individual may not accurately reflect that person’s unique context and often have limitations when applied to their particular situation. Consequently, the source of data plays a crucial role in determining their reliability and usefulness in medical practice, especially as a reference for interpreting laboratory results. It can be inferred that while big data is a strong resource for population-level references, it may not be as effective for individual-level insights. On the other hand, for an individual, it is not a realistic expectation to have a big dataset for most analytes. Therefore, statistical models for personalized laboratory medicine should be based on small- or middle-sized datasets. However, despite the small size, it can be speculated that, for individuals, models based on the individuals’ own data give better results than models based on big data obtained from the population. This is because in big datasets, individuals are accepted as a member of the big group, and each individual is usually represented by only a single measurement result. Therefore, the representativeness of big data for individuals is not as strong as that of small datasets obtained directly from individuals.

Although population data have limitations in representing individuals, it does not mean that they are useless in personalized medicine. While they should not be used directly to derive references for individuals, they can be used to develop general models for analytes, such as the patterns of ultradian, circadian, and infradian variations. These patterns can be applied to small datasets of individuals to indirectly estimate individual-specific references, particularly for personalized decision limit (prDL) and personalized action limit in diagnosis and treatment.

#### 4.3.1. Population-Based Reference Intervals for Personalized Laboratory Medicine

The popRIs of analytes are derived from population data and therefore should be used for populations. However, the critical point is that the population is not assessed based on popRI, and in reality, popRIs are used to evaluate individual data. Furthermore, the type of data collected to estimate the RI does not represent the physiological basis of RIs.

Investigations into RIs commenced in the 1960s [33,34,35,36,37,38,39], and since then, a consistent methodology has been applied, relying on the distribution of measurement results obtained from individual samples taken from a group of individuals for the estimation of RIs. Statistically, the popRI is estimated from the data of at least 120 individuals. Various statistical techniques depending on the distribution types of the data are being used to estimate the UL and LL of the popRI. The utilization of measurement results from a minimum of 120 subjects is necessary for the straightforward calculation of the UL, LL, and popRI. In the non-parametric estimation of RI, the measurement results are ranked from the lowest to the highest and the central 95% of the ranked data are accepted as the RIs. The LL and UL of the RI can be estimated through a straightforward calculation by excluding 5% of population from the ranked set of measurements: 2.5% from the lowest values and 2.5% from the highest values.

Despite advancements in statistical calculation techniques, there have been no substantial alterations in the theoretical framework governing the estimation of RIs, maintaining alignment with human physiology and the behavior of biomolecules within the human body. Due to the current theoretical framework of RIs being incompatible with the metabolism of molecules that fluctuate around an HSP, RIs based on conventional models have not been suitable for interpreting individual test results. Recently, we developed a model to estimate the popRI using data from a few reference individuals to determine the population set point (PSP) and a Gaussian combination of CV_I_, CV_G_, and CV_A_ to estimate the variation around the PSP [23]. The new model is more realistic and accounts for all types of variation in molecules observed within the population.

For an individual, the RI is based on the CV_P_ of the analytes. However, due to subclinical situations, the prRI based on repeated measurements may shift for some individuals. Therefore, using the popRI based on the PSP and biological variation (BV) data [23] can provide a reliable reference for prRIs and help prevent the estimation of extreme prRIs.

#### 4.3.2. Population-Based Decision Limits for Personalized Laboratory Medicine

Although RIs are commonly used by physicians to distinguish healthy subjects from diseased ones, the RI is not a diagnostic tool for all analytes and related diseases. For an analyte, RIs define a lower limit (2.5%) and an upper limit (97.5%) based on measurement results from samples obtained from healthy subjects. These limits cover 95% of the measurement results from healthy individuals and do not serve as cutoff values to distinguish healthy individuals from those with diseases. For diagnosing diseases, we need data from diseased patients rather than from healthy reference individuals. For an analyte, if the measurement result falls outside of the RI, it means that with a given probability (such as 95%), the result likely does not come from a healthy subject. However, to interpret this accurately, we need additional limits to make our decision. Therefore, the diagnosis of diseases relies on DLs, which are critical thresholds for analytes used in diagnosing diseases or making clinical decisions for specific situations, rather than on RIs.

Estimating the RI for an analyte is relatively easy because it is based on data from reference individuals. On the other hand, estimating the DL is challenging because it relies on data from patients with different clinical situations [6,40,41]. Population-based DL (popDL) can be estimated using data from test results of patients with specific clinical situations and diagnosed diseases. Since an analyte can be used in various clinical situations of a disease, there may be more than one popDL for an analyte. However, this is not the case for RIs, so an analyte has only one RI for a given age and sex. The DL is usually higher than the UL or lower than the LL of the RIs. However, in some clinical situations, it may be equal to the limits of the RIs but is not located within the RIs in any case. 

It can be questioned why, although an interval is used for healthy subjects, a similar interval is not used to interpret the measurement results of analytes in patients. In statistics, an interval such as an RI is estimated from a dataset that has a specific distribution type. Similar to healthy subjects, the measurement results of laboratory tests in patients have a distribution, but in contrast to healthy subjects, it is not rational to expect that data in pathological situations are normally or symmetrically distributed. Due to weak physiological control, the data are expected to be skewed toward the pathological side, and the range of fluctuation in measurement results around a theoretical set point is expected to be greater than the fluctuation observed in healthy subjects.

In patients, for an analyte, a single cut-off is often used for diagnosing diseases rather than an interval. If a higher level of the analyte is clinically significant, the lower limit of pathological data is used; conversely, if a lower level of the analyte is significant, the upper limit of pathological data is used to estimate the popDL. It should be noted that with RIs, the specificity of the laboratory test is evaluated (Equation (1)), while with DLs, the sensitivity of the laboratory test is assessed as formulated below (Figure 3).

(3) $$Sensitivity=\frac{TP}{TP+FN}$$

In personalized laboratory medicine, popDLs are essential, particularly for estimating prDLs. Since it is neither practical nor feasible to collect a sufficient dataset representing numerous clinical situations for deriving prDLs for an analyte, popDLs can be used as a reference to estimate prDLs through an indirect approach, as summarized below (see Section 4.4.2). 

### 4.4. Individuals’ Small Datasets and Personalized Laboratory Medicine

#### 4.4.1. Personalized Reference Intervals

From previous studies, it is known that the range of popRIs and prRIs are different for a measurand [22,42,43], and, therefore, it can be concluded that prRIs increase the specificity of laboratory tests by reducing the false positive rate, which is accurate for populations but not for individuals. The prRI can be estimated using an appropriate prediction interval model [44], based on individuals’ own data when they are medically in a steady state.

The classical approach is based on the HSP and the total variation around the HSP and can be formulated as shown below:

(4) $$prRI=HSP\;\pm k\times \;\sqrt{\frac{\left(n+1\right)}{n}x\;\left({SD}_{I/P}^{2}+{SD}_{A}^{2}\right)}$$

where *k* is the coverage factor, and its value depends on the type of statistical distribution. If *n* > 30, *k* is the z-value for 95%, which is 1.96. If *n* < 30, *k* is the t-table value for *n* − 1 degrees of freedom. *SD_I_*_/*P*_ represents the within-subject (obtained from a group of individuals) or within-person (obtained from individual’s own data) biological variation, and *SD_A_* is the analytical variation expressed in terms of standard deviation (SD).

It should be noted that if the variations in the amounts of analytes are given in terms of CV, such as CV_I/P_ and CV_A_, they must be converted to absolute values for the given concentration of the analytes before they can be combined with the HSP. Since the limits of prRIs are estimated using the BV data of measurands, the availability of reliable BV data [45] is essential for accurate prRIs. EFLM has launched the EFLM BV Database [46], which includes BV data for commonly requested measurands in laboratory medicine. These data are derived from meta-analyzed published BV studies [47,48,49,50] and from a multinational project, EuBIVAS, which has obtained high-quality BV data for numerous measurands [51,52,53,54].

#### 4.4.2. Personalized Decision Limits

Although popDLs are preferred by physicians to distinguish diseased subjects from healthy ones, for an individual, the diagnosis of diseases should be based on prDLs rather than popDLs. Hence, in practice, for the benefit of patients, personalizing DLs holds greater clinical significance than popDLs. However, estimating prDL for an analyte poses greater challenges.

In practice, it is not possible to collect sufficient data to derive the prDL for a measurand. However, this does not mean that prDL cannot be estimated. Although direct estimation is not feasible, indirect estimation can be performed using a model based on the relationship between popRI and popDL as detailed below:

Due to pathological processes during disease, it is assumed that the distribution of an analyte’s data shifts toward the pathological side. Therefore, the relative changes (popRC_DL_), i.e., the shift of population-based data from the popRI to the popDL, should be calculated as shown below [8]:

(5) $${popRC}_{DL}=\left|\frac{L_{popRI}-popDL}{L_{popRI}}\right|$$

where LpopRI represents the clinically significant limit of the popRI for the analyte. In the second step, the prDL can be estimated indirectly from popRC_DL_ as shown below.

(6) $$prDL=L_{prRI}\pm \;L_{prRI}\times {popRC}_{DL}=L_{prRI}\times \left(1\pm \;{popRC}_{DL}\right)$$

If the *DL* is located above the *UL*, then the mathematical sign should be “+”. However, if the *DL* is located below the *LL*, then the mathematical sign should be “−”. It can be concluded that, in comparison to the *popDL,* the *PrDL* may increase the sensitivity of laboratory tests by reducing the false negative rate, which is accurate for populations but not for individuals.

## 5. High-Dimensional Data and Personalized Laboratory Medicine

High-dimensional data (HDD) are characterized by datasets with a large number of variables or features, often exceeding the number of observations [55]. Mathematically, HDD can be represented as a matrix, as detailed below:

(7) $$X=\left[\begin{array}{cccc} x_{11} & x_{12} & \cdots & x_{1p}\\ x_{21} & x_{22} & \cdots & x_{2p}\\ \vdots & \vdots & \ddots & \vdots \\ x_{n1} & x_{n2} & \cdots & x_{np} \end{array}\right]$$

where *x_ij_* represents the value of the jth variable/feature for the ith measurement results. When the number of variables/features (p) is higher than the number of measurement results (*n*), the dataset is referred to as HDD.

Patients’ data, including medical history; physical examination findings; laboratory results; genetic information; radiological imaging such as MRIs, CT scans, and PET scans; medications; and more, stored in Electronic Health Records (EHRs), can be considered a high-dimensional dataset [56]. 

There are complex relationships among the biomolecules measured in medical laboratories, and their collective interactions create metabolism. Therefore, for an individual, laboratory data collected over time in a Laboratory Information System (LIS) can be considered HDD. Instead of relying on classical statistical approaches that evaluate single molecules or variables, statistical methods suited for HDD should be applied to interpret these data effectively [57].

Recently, wearable biosensors have become very popular and are used to monitor patients’ health, providing continuous data on various parameters that are crucial for managing chronic diseases such as diabetes mellitus, cardiovascular diseases, etc. [58,59,60,61]. These data contain a vast number of features for each patient.

The new step in disease diagnosis should be based on HDD rather than the evaluation of single properties of measurands. This approach requires the assessment of multidimensional data for personalized laboratory medicine. Such an evaluation should include time as a critical dimension and should assess progress over changing time intervals. AI-assisted tools are essential for implementing HDD in routine laboratory practice [62,63,64]. 

## 6. Artificial Intelligence and Machine Learning for Personalized Laboratory Medicine

At present, artificial intelligence (AI) has demonstrated significant promise in disease diagnosis. AI-assisted diagnostic tools can analyze radiological images like X-rays, CT scans, and MRIs, as well as evaluate medical history, examine symptoms, and process other disease-related information, helping physicians make quicker and more precise diagnoses [65,66,67,68,69,70]. 

AI has become an important tool in all steps of laboratory medicine and can be applied to all stages of total testing process [71]. Currently, AI can be used for human-based workflows including test utilization, error detection, result interpretation, genomics, and image analysis [72]. 

Personalized laboratory medicine can be regarded as high-dimensional laboratory medicine, incorporating multiple components such as the BV of analytes, chronobiology, precision laboratory medicine, and individualized statistical algorithms, among others [9]. For each individual, integrating and utilizing this complex information is challenging, making AI-assisted algorithms essential for disease diagnosis. Currently, AI-assisted algorithms are becoming important tools in general laboratory medicine [73,74,75,76,77]. The adoption of AI-assisted tools and algorithms will enable the evolution of conventional laboratory medicine into personalized laboratory medicine, enhancing personalized diagnosis and disease management.

## 7. Conclusions

Accurate diagnosis is essential for effective disease management. However, the diagnostic process involves numerous components and requires the integration of medical information from various sources, including patient history, examinations, radiological images, laboratory findings, etc. The references currently used to evaluate laboratory data are derived from population averages rather than individual-specific data. These references may not be accurate for individual patients, highlighting the need for a paradigm shift from conventional laboratory medicine to personalized laboratory medicine. 

Centuries ago, Nicolaus Copernicus revolutionized our understanding of the universe by shifting the center from the Earth to the Sun [78]. Similarly, it is now time to shift the focus of medicine from population averages to individual-specific data. In modern medicine, patients should be at the center of disease management. To facilitate this paradigm shift, we must leverage personalized statistical algorithms and artificial intelligence.

## Data Availability

No new data were created or analyzed in this study. Data sharing is not applicable to this article.
